# (4-Bromo­phen­yl)(2,7-dimeth­oxy-1-naphth­yl)methanone

**DOI:** 10.1107/S1600536810042662

**Published:** 2010-10-23

**Authors:** Yuichi Kato, Atsushi Nagasawa, Takehiro Tsumuki, Akiko Okamoto, Noriyuki Yonezawa

**Affiliations:** a Department of Organic and Polymer Materials Chemistry, Tokyo University of Agriculture & Technology, 2-24-16 Naka-machi, Koganei, Tokyo 184-8588, Japan

## Abstract

In the title compound, C_19_H_15_BrO_3_, the dihedral angle between the naphthalene ring system and the benzene ring is 72.02 (9)°. The bridging carbonyl C—C(=O)—C plane makes dihedral angles of 70.88 (10) and 1.87 (12)°, respectively, with the naphthalene ring system and the benzene ring. In the crystal, two types of weak inter­molecular C—H⋯O inter­actions and a short Br⋯C contact [3.345 (2) Å] are observed.

## Related literature

For electrophilic aromatic substitution of naphthalene deriva­tives, see: Okamoto & Yonezawa (2009 ▶). For the structures of closely related compounds, see: Hijikata *et al.*, 2010 ▶); Kato, Nagasawa, Hijikata *et al.* (2010 ▶); Kato, Nagasawa, Kataoka *et al.* (2010 ▶); Muto *et al.* (2010 ▶); Watanabe, Muto *et al.* (2010 ▶); Watanabe, Nakaema *et al.* (2010 ▶).
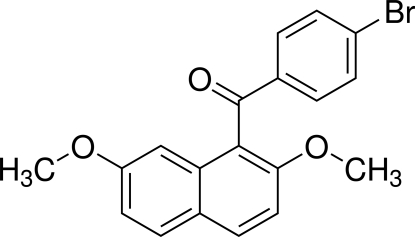


## Experimental

### 

#### Crystal data

C_19_H_15_BrO_3_
*M* *_r_* = 371.22
Orthorhombic, 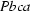

*a* = 6.58278 (12) Å
*b* = 16.1134 (3) Å
*c* = 30.2750 (6) Å
*V* = 3211.30 (10) Å^3^
*Z* = 8
Cu *K*α radiation
μ = 3.60 mm^−1^
*T* = 193 K
0.60 × 0.60 × 0.20 mm

#### Data collection

Rigaku R-AXIS RAPID diffractometer
Absorption correction: numerical (*NUMABS*; Higashi, 1999 ▶) *T*_min_ = 0.161, *T*_max_ = 0.533
52243 measured reflections
2922 independent reflections
2724 reflections with *I* > 2σ(*I*)
*R*_int_ = 0.033

#### Refinement

*R*[*F* ^2^ > 2σ(*F* ^2^)] = 0.033
*wR*(*F* ^2^) = 0.082
*S* = 1.06
2922 reflections
211 parameters
H-atom parameters constrained
Δρ_max_ = 0.73 e Å^−3^
Δρ_min_ = −0.67 e Å^−3^

### 

Data collection: *PROCESS-AUTO* (Rigaku, 1998 ▶); cell refinement: *PROCESS-AUTO*; data reduction: *CrystalStructure* (Rigaku/MSC, 2004 ▶); program(s) used to solve structure: *SIR2004* (Burla *et al.*, 2005 ▶); program(s) used to refine structure: *SHELXL97* (Sheldrick, 2008 ▶); molecular graphics: *ORTEPIII* (Burnett & Johnson, 1996 ▶); software used to prepare material for publication: *SHELXL97*.

## Supplementary Material

Crystal structure: contains datablocks global, I. DOI: 10.1107/S1600536810042662/is2618sup1.cif

Structure factors: contains datablocks I. DOI: 10.1107/S1600536810042662/is2618Isup2.hkl

Additional supplementary materials:  crystallographic information; 3D view; checkCIF report

## Figures and Tables

**Table 1:** Hydrogen-bond geometry (Å, °)

*D*—H⋯*A*	*D*—H	H⋯*A*	*D*⋯*A*	*D*—H⋯*A*
C6—H6⋯O1^i^	0.95	2.57	3.372 (3)	142
C17—H17⋯O2^ii^	0.95	2.57	3.407 (3)	148

Symmetry codes: (i) 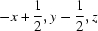; (ii) 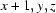.

**Fig. 1.**
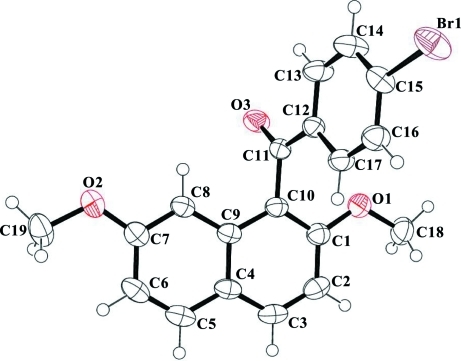
Molecular structure with displacement ellipsoids at 50% probability.

**Fig. 2.**
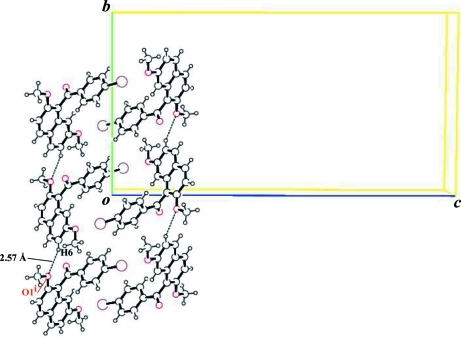
Intermolecular C6—H6···O1 interactions, viewed along the a axis [symmetry code: (i) -x + 1/2, y - 1/2, z].

**Fig. 3.**
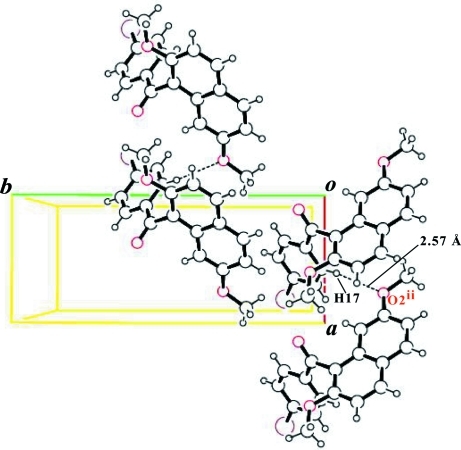
Intermolecular C17—H17···O2 interactions, viewed along the c axis [symmetry code: (ii) x + 1, y, z].

**Fig. 4.**
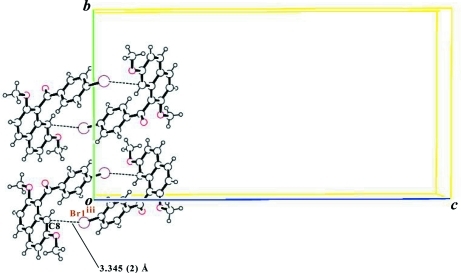
Intermolecular interactions between bromine atom Br1 and naphthalene ring carbon atom C8, viewed along the a axis [symmetry code: (iii) -x + 1, -y, -z]. .

## supplementary crystallographic information

### Comment

In the course of our study on electrophilic aromatic aroylation of 2,7-dimethoxynaphthalene, *peri*-aroylnaphthalene compounds have proven to be formed regioselectively with the aid of suitable acidic mediators (Okamoto & Yonezawa, 2009). Recently, we have reported the crystal structures of several 1,8-diaroylated naphthalene homologues exemplified by 1,8-bis(4-methylbenzoyl)-2,7-dimethoxynaphthalene (Muto *et al.*, 2010). The aroyl groups at the 1,8-positions of the naphthalene rings in these compounds are twistedly connected in an almost perpendicular fashion, but the benzene ring moieties of the aroyl groups tilt slightly toward the *exo* sides of the naphthalene rings. In this course, the crystal structures of 1-monoaroylated naphthalene compounds and the *β*-isomers of 3-monoaroylated compounds have been also clarified such as 2-(2,7-dimethoxy-1-naphthoyl)benzoic acid (Hijikata *et al.*, 2010), (2,7-dimethoxynaphthalen-1-yl)(phenyl)methanone (Kato, Nagasawa, Hijikata *et al.*, 2010) and (3,6-dimethoxy-2-naphthyl)(4-fluorophenyl)methanone (Watanabe, Muto *et al.*, 2010). The former compounds have revealed to have essentially the same non-coplanar structure with the 1,8-diaroylated naphthalenes. On the other hand, presence of bromo groups in these compounds are shown to demonstrate somewhat different as displayed for aroylated naphthalene homologues bearing bromo group, *i.e.*, bis(4-bromophenyl)(2,7-dimethoxynaphthalene-1,8-diyl)dimethanone (Watanabe, Nakaema *et al.*, 2010) and (4-bromophenyl)(3,6-dimethoxy-2-naphthyl)methanone The 4-bromophenyl group is out of the plane of the naphthalene ring. (Kato, Nagasawa, Kataoka *et al.*, 2010). In the crystal structures, the dihedral angles between naphthalene and bromophenyl rings are demonstrated to have rather small compared to the corresponding without bromo group compounds. As a part of the course of our continuous study on the molecular structures of these kinds of homologous molecules, the crystal structure of title compound, a 1-bromobenzoylated naphthalene derivative, is discussed in this paper.

The molecular structure of the title compound is displayed in Fig. 1. The 4-bromophenyl group is out of the plane of the naphthalene ring. The dihedral angle between the best planes of the bromophenyl ring (C12—C17) and the naphthalene ring (C1—C10) is 72.02 (9)°. The carbonyl group and the 4-bromophenyl group have almost coplanar configuration [O3—C11—C12—C13 torsion angle = 2.5 (3)°]. On the other hand, the carbonyl group makes torsion angle of 70.3 (3)° with the naphthalene ring plane.

In the crystal structure, the molecular packing of the title compound is stabilized mainly by van der Waals interactions. The crystal packing is additionally stabilized by intermolecular C—H···O hydrogen bonding between the oxygen atom (O1) of the 2-methoxy group and one hydrogen atom (H6) of the naphthalene ring of the adjacent molecule along the *a* axis (C6—H6···O1^i^; Fig. 2 and Table 1). Moreover, there is also intermolecular C—H···O hydrogen bonding between the oxygen atom (O2) of the 7-methoxy group and one hydrogen atom (H17) of the 4-bromophenyl group of the adjacent molecule along the *c* axis (C17—H17···O2^ii^; Fig. 3 and Table 1). Furthermore, an intermolecular interaction between the bromine atom and the naphthalene ring carbon [C8···Br1^iii^ = 3.345 (2) Å; (iii) -*x* + 1, -*y*, -*z*] is observed (Fig. 4).

### Experimental

To a 100 ml flask, 4-bromobenzoyl chloride (11 mmol, 2.403 g), aluminium chloride (13.3 mmol, 1.769 g) and methylene chloride (25 ml) were placed and stirred at 273 K. To the reaction mixture thus obtained, 2,7-dimethoxynaphthalene (9.9 mmol, 1.869 g) and methylene chloride (25 ml) were added. After the reaction mixture was stirred at 273 K for 6 h, it was poured into ice-cold water (10 ml). The aqueous layer was extracted with CHCl_3_ (10 ml × 3). The combined extracts were washed with 2 *M* aqueous NaOH followed by washing with brine. The organic layers thus obtained were dried over anhydrous MgSO_4_. The solvent was removed under reduced pressure to give cake. The crude product was purified by recrystallization from ethanol (57% yield). Colorless platelet single crystals suitable for X-ray diffraction were obtained by repeated crystallization from ethanol.

Spectroscopic Data:

^1^H NMR δ (300 MHz, CDCl_3_); 3.73 (3*H*, s), 3.78 (3*H*, s), 6.78 (1*H*, d, *J* = 2.4 Hz), 7.02 (1*H*, dd, *J* = 2.4, 9.0 Hz), 7.15 (1*H*, d, *J* = 9.3 Hz), 7.56 (2*H*, d, *J* = 8.7 Hz), 7.69–7.74 (3*H*, m), 7.87 (1*H*, d, *J* = 9.0 Hz). ^13^C NMR δ (75 MHz, CDCl_3_); 54.90, 55.95, 101.73, 109.85, 116.84, 120.69, 124.12, 128.29, 129.57, 130.74, 131.16, 131.63, 132.74, 136.71, 154.87, 158.76, 196.67. IR (KBr); 1667 (C═O), 1626, 1572, 1513 (Ar, naphthalene) cm^-1^. HRMS (*m/z*); [*M* + Na]^+^ Calcd for C_19_H_15_O_3_BrNa, 393.0102; found, 393.0106. m.p. = 405.2–408.7 K

### Refinement

All H atoms were found in a difference map and were subsequently refined as riding atoms, with C—H = 0.95 (aromatic) and 0.98 (methyl) Å, and with *U*_iso_(H) = 1.2*U*_eq_(C).

### Crystal data

**Table:**

C_19_H_15_BrO_3_	*D*_x_ = 1.536 Mg m^−^^3^
*M**_r_* = 371.22	Melting point = 405.2–408.7 K
Orthorhombic, *P**b**c**a*	Cu *K*α radiation, λ = 1.54187 Å
Hall symbol: -P 2ac 2ab	Cell parameters from 50764 reflections
*a* = 6.58278 (12) Å	θ = 3.1–68.2°
*b* = 16.1134 (3) Å	µ = 3.60 mm^−^^1^
*c* = 30.2750 (6) Å	*T* = 193 K
*V* = 3211.30 (10) Å^3^	Platelet, colorless
*Z* = 8	0.60 × 0.60 × 0.20 mm
*F*(000) = 1504	

### Data collection

**Table:**

Rigaku R-AXIS RAPID diffractometer	2922 independent reflections
Radiation source: rotating anode	2724 reflections with *I* > 2σ(*I*)
graphite	*R*_int_ = 0.033
Detector resolution: 10.00 pixels mm^-1^	θ_max_ = 68.3°, θ_min_ = 5.5°
ω scans	*h* = −7→7
Absorption correction: numerical (*NUMABS*; Higashi, 1999)	*k* = −19→19
*T*_min_ = 0.161, *T*_max_ = 0.533	*l* = −36→36
52243 measured reflections	

### Refinement

**Table:**

Refinement on *F*^2^	Secondary atom site location: difference Fourier map
Least-squares matrix: full	Hydrogen site location: inferred from neighbouring sites
*R*[*F*^2^ > 2σ(*F*^2^)] = 0.033	H-atom parameters constrained
*wR*(*F*^2^) = 0.082	*w* = 1/[σ^2^(*F*_o_^2^) + (0.0352*P*)^2^ + 2.8492*P*] where *P* = (*F*_o_^2^ + 2*F*_c_^2^)/3
*S* = 1.06	(Δ/σ)_max_ < 0.001
2922 reflections	Δρ_max_ = 0.73 e Å^−^^3^
211 parameters	Δρ_min_ = −0.67 e Å^−^^3^
0 restraints	Extinction correction: *SHELXL97* (Sheldrick, 2008), Fc^*^=kFc[1+0.001xFc^2^λ^3^/sin(2θ)]^-1/4^
Primary atom site location: structure-invariant direct methods	Extinction coefficient: 0.00189 (8)

### Special details

**Table:**

Geometry. All e.s.d.'s (except the e.s.d. in the dihedral angle between two l.s. planes) are estimated using the full covariance matrix. The cell e.s.d.'s are taken into account individually in the estimation of e.s.d.'s in distances, angles and torsion angles; correlations between e.s.d.'s in cell parameters are only used when they are defined by crystal symmetry. An approximate (isotropic) treatment of cell e.s.d.'s is used for estimating e.s.d.'s involving l.s. planes.
Refinement. Refinement of *F*^2^ against ALL reflections. The weighted *R*-factor *wR* and goodness of fit *S* are based on *F*^2^, conventional *R*-factors *R* are based on *F*, with *F* set to zero for negative *F*^2^. The threshold expression of *F*^2^ > σ(*F*^2^) is used only for calculating *R*-factors(gt) *etc*. and is not relevant to the choice of reflections for refinement. *R*-factors based on *F*^2^ are statistically about twice as large as those based on *F*, and *R*- factors based on ALL data will be even larger.

### Fractional atomic coordinates and isotropic or equivalent isotropic displacement parameters (Å^2^)

**Table:**

	*x*	*y*	*z*	*U*_iso_*/*U*_eq_	
Br1	0.81329 (5)	0.125182 (16)	0.029041 (9)	0.05922 (14)	
O1	0.5963 (3)	0.05334 (10)	−0.18778 (5)	0.0474 (4)	
O2	−0.2447 (3)	−0.17865 (10)	−0.10564 (6)	0.0520 (4)	
O3	0.1223 (2)	0.08709 (10)	−0.13828 (6)	0.0487 (4)	
C1	0.4972 (3)	−0.02136 (13)	−0.18754 (7)	0.0374 (5)	
C2	0.5580 (4)	−0.08984 (15)	−0.21337 (7)	0.0435 (5)	
H2	0.6741	−0.0861	−0.2319	0.052*	
C3	0.4479 (4)	−0.16140 (14)	−0.21143 (7)	0.0431 (5)	
H3	0.4914	−0.2078	−0.2283	0.052*	
C4	0.2719 (4)	−0.16887 (13)	−0.18527 (6)	0.0366 (5)	
C5	0.1554 (4)	−0.24241 (13)	−0.18287 (7)	0.0428 (5)	
H5	0.1968	−0.2893	−0.1996	0.051*	
C6	−0.0143 (4)	−0.24819 (13)	−0.15728 (7)	0.0438 (5)	
H6	−0.0894	−0.2985	−0.1562	0.053*	
C7	−0.0776 (3)	−0.17878 (13)	−0.13235 (7)	0.0388 (5)	
C8	0.0304 (3)	−0.10607 (12)	−0.13367 (7)	0.0348 (4)	
H8	−0.0151	−0.0598	−0.1170	0.042*	
C9	0.2085 (3)	−0.09892 (12)	−0.15950 (6)	0.0322 (4)	
C10	0.3289 (3)	−0.02594 (12)	−0.16062 (6)	0.0331 (4)	
C11	0.2740 (3)	0.04610 (12)	−0.13110 (7)	0.0336 (4)	
C12	0.4065 (3)	0.06341 (12)	−0.09228 (6)	0.0322 (4)	
C13	0.3595 (4)	0.12986 (14)	−0.06479 (8)	0.0458 (6)	
H13	0.2440	0.1632	−0.0710	0.055*	
C14	0.4791 (4)	0.14768 (15)	−0.02861 (8)	0.0514 (6)	
H14	0.4468	0.1931	−0.0099	0.062*	
C15	0.6459 (4)	0.09888 (14)	−0.01995 (7)	0.0410 (5)	
C16	0.6948 (4)	0.03213 (14)	−0.04623 (8)	0.0430 (5)	
H16	0.8101	−0.0012	−0.0397	0.052*	
C17	0.5735 (3)	0.01439 (13)	−0.08224 (7)	0.0385 (5)	
H17	0.6048	−0.0320	−0.1004	0.046*	
C18	0.7728 (4)	0.06171 (18)	−0.21481 (8)	0.0534 (6)	
H18A	0.8301	0.1174	−0.2111	0.064*	
H18B	0.7357	0.0533	−0.2458	0.064*	
H18C	0.8738	0.0201	−0.2061	0.064*	
C19	−0.3709 (4)	−0.25055 (15)	−0.10462 (10)	0.0570 (7)	
H19A	−0.4878	−0.2403	−0.0853	0.068*	
H19B	−0.2930	−0.2978	−0.0933	0.068*	
H19C	−0.4188	−0.2630	−0.1345	0.068*	

### Atomic displacement parameters (Å^2^)

**Table:**

	*U*^11^	*U*^22^	*U*^33^	*U*^12^	*U*^13^	*U*^23^
Br1	0.0793 (2)	0.04578 (18)	0.05256 (19)	−0.00079 (13)	−0.02664 (14)	−0.00441 (11)
O1	0.0446 (9)	0.0476 (9)	0.0499 (9)	−0.0074 (8)	0.0127 (7)	−0.0077 (7)
O2	0.0498 (9)	0.0384 (8)	0.0678 (11)	−0.0113 (8)	0.0073 (9)	−0.0016 (8)
O3	0.0400 (9)	0.0376 (8)	0.0684 (11)	0.0100 (7)	−0.0141 (8)	−0.0134 (8)
C1	0.0381 (11)	0.0380 (11)	0.0361 (10)	0.0026 (9)	−0.0025 (9)	−0.0024 (8)
C2	0.0434 (13)	0.0516 (13)	0.0356 (11)	0.0099 (11)	0.0031 (9)	−0.0046 (10)
C3	0.0537 (14)	0.0394 (12)	0.0362 (11)	0.0153 (10)	−0.0024 (10)	−0.0089 (9)
C4	0.0465 (12)	0.0311 (10)	0.0323 (10)	0.0095 (9)	−0.0099 (9)	−0.0034 (8)
C5	0.0586 (15)	0.0302 (11)	0.0397 (11)	0.0087 (10)	−0.0109 (10)	−0.0055 (9)
C6	0.0570 (14)	0.0272 (10)	0.0474 (12)	−0.0015 (10)	−0.0151 (11)	0.0008 (9)
C7	0.0410 (12)	0.0347 (11)	0.0408 (11)	−0.0001 (9)	−0.0073 (9)	0.0025 (9)
C8	0.0383 (11)	0.0290 (10)	0.0370 (10)	0.0030 (9)	−0.0049 (9)	−0.0024 (8)
C9	0.0363 (11)	0.0288 (10)	0.0315 (10)	0.0064 (8)	−0.0082 (8)	−0.0010 (8)
C10	0.0341 (11)	0.0313 (10)	0.0337 (10)	0.0052 (8)	−0.0035 (8)	−0.0036 (8)
C11	0.0325 (11)	0.0273 (10)	0.0409 (11)	0.0001 (8)	0.0012 (9)	−0.0010 (8)
C12	0.0343 (10)	0.0264 (9)	0.0359 (10)	0.0003 (8)	0.0025 (8)	0.0003 (8)
C13	0.0521 (14)	0.0371 (12)	0.0482 (13)	0.0159 (10)	−0.0060 (11)	−0.0082 (9)
C14	0.0690 (17)	0.0387 (12)	0.0464 (13)	0.0149 (12)	−0.0109 (12)	−0.0121 (10)
C15	0.0539 (14)	0.0320 (10)	0.0372 (11)	−0.0042 (10)	−0.0090 (10)	0.0008 (9)
C16	0.0441 (13)	0.0392 (12)	0.0457 (12)	0.0076 (10)	−0.0060 (10)	−0.0015 (10)
C17	0.0401 (12)	0.0339 (11)	0.0416 (11)	0.0063 (9)	−0.0001 (9)	−0.0048 (9)
C18	0.0423 (13)	0.0665 (16)	0.0515 (13)	−0.0103 (12)	0.0106 (11)	−0.0061 (12)
C19	0.0532 (15)	0.0431 (13)	0.0747 (17)	−0.0137 (12)	−0.0049 (13)	0.0122 (12)

### Geometric parameters (Å, °)

**Table:**

Br1—C15	1.896 (2)	C8—H8	0.9500
O1—C1	1.369 (3)	C9—C10	1.419 (3)
O1—C18	1.428 (3)	C10—C11	1.509 (3)
O2—C7	1.365 (3)	C11—C12	1.490 (3)
O2—C19	1.426 (3)	C12—C17	1.387 (3)
O3—C11	1.217 (3)	C12—C13	1.391 (3)
C1—C10	1.378 (3)	C13—C14	1.380 (3)
C1—C2	1.410 (3)	C13—H13	0.9500
C2—C3	1.363 (3)	C14—C15	1.375 (4)
C2—H2	0.9500	C14—H14	0.9500
C3—C4	1.409 (3)	C15—C16	1.376 (3)
C3—H3	0.9500	C16—C17	1.381 (3)
C4—C5	1.413 (3)	C16—H16	0.9500
C4—C9	1.433 (3)	C17—H17	0.9500
C5—C6	1.363 (3)	C18—H18A	0.9800
C5—H5	0.9500	C18—H18B	0.9800
C6—C7	1.412 (3)	C18—H18C	0.9800
C6—H6	0.9500	C19—H19A	0.9800
C7—C8	1.371 (3)	C19—H19B	0.9800
C8—C9	1.414 (3)	C19—H19C	0.9800
			
C1—O1—C18	118.30 (18)	O3—C11—C10	120.55 (19)
C7—O2—C19	118.76 (19)	C12—C11—C10	118.10 (17)
O1—C1—C10	115.70 (18)	C17—C12—C13	118.9 (2)
O1—C1—C2	123.3 (2)	C17—C12—C11	122.02 (18)
C10—C1—C2	121.0 (2)	C13—C12—C11	119.05 (19)
C3—C2—C1	119.2 (2)	C14—C13—C12	120.5 (2)
C3—C2—H2	120.4	C14—C13—H13	119.7
C1—C2—H2	120.4	C12—C13—H13	119.7
C2—C3—C4	122.3 (2)	C15—C14—C13	119.2 (2)
C2—C3—H3	118.9	C15—C14—H14	120.4
C4—C3—H3	118.9	C13—C14—H14	120.4
C3—C4—C5	123.16 (19)	C14—C15—C16	121.6 (2)
C3—C4—C9	118.6 (2)	C14—C15—Br1	119.02 (17)
C5—C4—C9	118.2 (2)	C16—C15—Br1	119.40 (18)
C6—C5—C4	122.1 (2)	C15—C16—C17	118.9 (2)
C6—C5—H5	118.9	C15—C16—H16	120.6
C4—C5—H5	118.9	C17—C16—H16	120.6
C5—C6—C7	119.4 (2)	C16—C17—C12	120.88 (19)
C5—C6—H6	120.3	C16—C17—H17	119.6
C7—C6—H6	120.3	C12—C17—H17	119.6
O2—C7—C8	115.71 (19)	O1—C18—H18A	109.5
O2—C7—C6	123.7 (2)	O1—C18—H18B	109.5
C8—C7—C6	120.5 (2)	H18A—C18—H18B	109.5
C7—C8—C9	121.04 (19)	O1—C18—H18C	109.5
C7—C8—H8	119.5	H18A—C18—H18C	109.5
C9—C8—H8	119.5	H18B—C18—H18C	109.5
C8—C9—C10	122.95 (18)	O2—C19—H19A	109.5
C8—C9—C4	118.61 (19)	O2—C19—H19B	109.5
C10—C9—C4	118.44 (19)	H19A—C19—H19B	109.5
C1—C10—C9	120.52 (18)	O2—C19—H19C	109.5
C1—C10—C11	120.11 (19)	H19A—C19—H19C	109.5
C9—C10—C11	119.35 (18)	H19B—C19—H19C	109.5
O3—C11—C12	121.32 (18)		
			
C18—O1—C1—C10	179.6 (2)	O1—C1—C10—C11	−4.8 (3)
C18—O1—C1—C2	−1.4 (3)	C2—C1—C10—C11	176.16 (19)
O1—C1—C2—C3	−179.1 (2)	C8—C9—C10—C1	−177.79 (19)
C10—C1—C2—C3	−0.1 (3)	C4—C9—C10—C1	3.1 (3)
C1—C2—C3—C4	1.6 (3)	C8—C9—C10—C11	3.8 (3)
C2—C3—C4—C5	179.9 (2)	C4—C9—C10—C11	−175.36 (18)
C2—C3—C4—C9	−0.7 (3)	C1—C10—C11—O3	111.3 (2)
C3—C4—C5—C6	179.9 (2)	C9—C10—C11—O3	−70.3 (3)
C9—C4—C5—C6	0.5 (3)	C1—C10—C11—C12	−70.8 (3)
C4—C5—C6—C7	0.2 (3)	C9—C10—C11—C12	107.6 (2)
C19—O2—C7—C8	176.4 (2)	O3—C11—C12—C17	176.3 (2)
C19—O2—C7—C6	−4.0 (3)	C10—C11—C12—C17	−1.6 (3)
C5—C6—C7—O2	−179.7 (2)	O3—C11—C12—C13	−2.5 (3)
C5—C6—C7—C8	−0.1 (3)	C10—C11—C12—C13	179.6 (2)
O2—C7—C8—C9	178.88 (18)	C17—C12—C13—C14	1.2 (4)
C6—C7—C8—C9	−0.7 (3)	C11—C12—C13—C14	−179.9 (2)
C7—C8—C9—C10	−177.67 (19)	C12—C13—C14—C15	−0.1 (4)
C7—C8—C9—C4	1.4 (3)	C13—C14—C15—C16	−0.7 (4)
C3—C4—C9—C8	179.24 (18)	C13—C14—C15—Br1	178.6 (2)
C5—C4—C9—C8	−1.3 (3)	C14—C15—C16—C17	0.3 (4)
C3—C4—C9—C10	−1.6 (3)	Br1—C15—C16—C17	−178.97 (17)
C5—C4—C9—C10	177.83 (18)	C15—C16—C17—C12	0.9 (3)
O1—C1—C10—C9	176.75 (18)	C13—C12—C17—C16	−1.6 (3)
C2—C1—C10—C9	−2.3 (3)	C11—C12—C17—C16	179.5 (2)

### Hydrogen-bond geometry (Å, °)

**Table:**

*D*—H···*A*	*D*—H	H···*A*	*D*···*A*	*D*—H···*A*
C6—H6···O1^i^	0.95	2.57	3.372 (3)	142
C17—H17···O2^ii^	0.95	2.57	3.407 (3)	148

Symmetry codes: (i) −*x*+1/2, *y*−1/2, *z*; (ii) *x*+1, *y*, *z*.
